# Obstetrics, and Diseases of Women and Children

**Published:** 1860-11

**Authors:** Wm. B. Atkinson

**Affiliations:** Lecturer on Obstetrics, and Diseases of Women and Children


### OBSTETRICS, AND DISEASES OF WOMEN AND CHILDREN.

BY WM. B. ATKINSON, M.D.,
LECTURER ON OBSTETRICS, AND DISEASES OF WOMEN AND CHILDREN.

### I.—*A New Method of Version in Abnormal Labor.* By J. BRAXTON HICKS, M D., F.L.S., etc., London.

One of the most important facts in regard to version is, that if we place the fœtus in utero in a transverse position and abdomen downward, its knees will be opposite the os, and may be hooked down by a finger, if the dilation is sufficient. Next, it is rare that one side or the other is not more dependent, and, consequently, one knee is within reach of the finger. Again, the shape of the cavity of the fully developed uterus being oval, with the long axis placed vertically, the tendency of the slight pains toward the end of pregnancy is to place the longer axis of the fœtus in a corresponding direction, so that if the fœtus be transverse, a slight preponderance of force in either direction will determine the ultimate presentation. His method is conducted thus: Suppose the simplest condition, where the uterus is passive, the membranes unbroken, the liquor amnii plentiful, the os expanded sufficiently to detect the presentation, which is cephalic, and the occiput to the left side: the patient is in the ordinary position, the trunk curved forward to relax the abdominal muscles. Introduce the left hand into the vagina, so as to fairly touch the head of the child. Having passed one or two fingers within the cervix, and resting them on the head, place the right hand on the left side of the breech at the fundus uteri. Employ gentle pressure and slight impulsive movements on the fundus toward the right side, and simultaneously on the head toward the left iliac fossa. In a very short time the head will be found to rise and the breech to descend; the fœtus is now transverse, the knee opposite the os, the membranes may be ruptured, and the labor terminated as usual in turning. In obedience to the law above stated, when the fœtus is placed transversely, a slight impulse will determine the final position of the head. When the leg is seized, it is therefore advisable to place the right hand beneath the head in its new position on the left side, and gently press it toward the fundus. The same law renders it very easy to convert the cephalic, shoulder, neck, or even natural transverse, into a breech case. In either of these, it is merely necessary to push up the head, and, removing the left hand from the vagina, without bringing down the knee or foot, place it on the breech in the right iliac fossa, so as to depress it into the cavity of the pelvis. No extra force should be used. for it will be found to obey a very gentle persuasion. In other presentations from the one supposed, the only changes required will be to make the pressure and impulses in opposite directions. When the exact position cannot be made out, treat it as the first or fourth. If the liquor amnii has escaped, though the operation is more difficult, yet even then it is easily managed, and if the *uterus be too active, suspend it for a moment by chloroform or opium.*

By this method, we avoid the addition of the hand, and perhaps arm, to the contents of the uterus, and the irritation, present and future, caused by it; the entry of air within the uterine cavity; liability to rupture of the uterus; and the pain and distress felt in the ordinary plan. Another advantage is, that the membranes need not be ruptured. This method is peculiarly applicable to all malpositions; in puerperal convulsions, in narrowness of the brim of the pelvis, it saves the pressure upon the os uteri against the projecting parts, and in cases of placenta prævia.

The author then relates a number of cases, in which this plan was successfully employed.—(*Lancet*, July 14th, 21st, 1860.)

II.—*On the Delivery of the Placenta, particularly in Abortions in Early Pregnancy.* By L. D. Sheetz, M.D., Indiana.

This subject is one of the most important which can engage the attention of the accoucheur, and requires to be perfectly understood by him prior to his entrance into the lying-in chamber. Cases frequently occur where abortion has taken place, the embryo has been extruded, excessive hemorrhage follows, syncope approaches, and no time is to be lost. The medical attendant has no time to consult authorities; it is required of him to act quickly and well. At full term the placenta, as a general rule, is easily managed; but when adhesion has taken place, accompanied by hour-glass contractions, much difficulty is experienced.

The *immediate* delivery of the placenta is required, particularly in abortions with much hemorrhage. In nearly all cases at term, the after-birth is found, after the delivery of the child, lying in the vagina, and may be extracted by the use of gentle traction. If the uterus has not sufficiently contracted to throw it off, gentle frictions should be made over the abdomen, which will assist in arousing the uterus, causing it to expel its contents, and thus guard against concealed hemorrhage. When the placenta is adherent, the womb still retains to a certain extent its elongated form; the circular fibres are contracted, while the longitudinal fibres remain in a flaccid condition. In this state hemorrhage, to an exhausting extent, will speedily occur. There is no use in waiting for nature to relieve such a case; but the physician must at once resort to manual delivery Though cases have been reported where the placenta was so closely adherent as to defy separation even after death, yet these instances are rare. In cases of abortion, during the first month, the uterus being but slightly developed, its vessels are consequently small, and there is very little bleeding—besides, there is as yet no placenta; but after the second or third months, excessive hemorrhage generally attends these cases. When the placenta cannot be felt by the touch at the os, the finger should be passed in, so as to traverse the whole interior of the womb, when, if a placenta be found, efforts should at once be made to remove it. The idea seems to be prevalent, that it can only be removed in early abortions, if at all, by the use of some instrument, as the forceps or hook, and that when these fail, recourse must be had to the tampon, ergot, etc., to arrest the hemorrhage; the after-birth being left to the process of decomposition. Dr. Sheetz is persuaded that the tampon is often a source of mischief, being employed very frequently before any attempt has been made to extract the placenta.

After referring to the views of Meigs, Chailly, Ramsbotham, and other writers on this subject, Dr. Sheetz gives his mode of procedure: "My method is to remove the placenta with the index finger, on which I always keep a tolerably long nail for the purpose of separating the after-birth from the womb. The nail should not be too long, or it will not be sufficiently firm, and may bend backward while using it. Push the uterus as low down in the pelvis as possible, and retain it there with the left hand, while you introduce the index finger of the right hand into the cavity, and peal off the placenta and remove it. Sometimes it is a little difficult to separate with a single finger, and I do not get quite all removed; but it is so much broken up as to arrest the violence of the hemorrhage, and render the tampon, etc., unnecessary; in a few days the remainder will come away piecemeal. The detachment of the placenta causes considerable pain, and you are sometimes obliged to desist a few minutes, without removing the finger to let the patient have a little rest; but I have never seen any bad results follow the operation."—(*Cleveland Med. Gaz.*, July, 1860.)

III.—*A Case of Rupture of the Uterus.* By JOHN H. ALDRIDGE, M.D., Southampton.

Mrs. S., aged forty-one, tall, robust, never any illness, had been married eight years and given birth to seven children, two of whom were born dead. She was taken in labor at 12 P.M., March thirtieth, with strong uterine contractions. On the next day, (the thirty-first,) at 3 P.M., suddenly, after a very violent pain, she felt something give way in her abdomen, with a snap, and the passage of a clot of blood from the vagina. From that time there was a slight red discharge, and no more proper labor-pains. She went about her household duties all that day, and with occasional severe pain at the lower part of the abdomen, until the fifth of April, when the discharge became fetid, and the abdomen much distended, and painful. On the sixth, the odor of the discharge had become horribly offensive, of a blackish red, and watery. The pulse was quick, but full and strong, the abdomen tympanitic, so that no part of the child could be felt. On examination, the os easily admitted the passage of two fingers, though no portion of the child could be reached. The diagnosis was rupture of the uterus, and the operation of turning was resorted to, with the view of bringing the child away.

At 3 P.M. of the sixth, the left hand was passed quite into the uterus, the membranes were ruptured, and a full-sized male child extracted without difficulty. It was putrid, and must have been dead for several days. The placenta was brought away in a similar state. The patient sank gradually, and died on the eleventh of April, eleven days after the supposed period of rupture.

The autopsy showed the peritoneum containing a considerable quantity of the same kind of fluid as that discharged from the vagina. The intestines were adherent, and gave evidence of the existence of severe and recent peritonitis. The fundus uteri protruded above the symphysis pubis, and, on washing away the blackish-red fluid, the rupture was immediately seen. It extended from the right extremity of the fundus obliquely through the whole body and cervix of the organ, and for some distance into the upper wall of the vagina. There was no unusual prominence or roughness of the pelvic bones which could have predisposed to this accident. In all likelihood, the rupture, which occurred on the thirty-first of March, was enlarged to the extent noticed during the operation of extracting the child. It is difficult to assign any reason for the severe injury to the womb in this case, as no predisposing causes could be found. The pelvis was of normal dimensions, there was no history of a fall or injury during the pregnancy, and, so far as can be judged from the woman's account of the labor, the uterus had not been subjected for any unusual length of time to great pressure or attrition from its own expulsive efforts. The circumstance of her having lived so long after the accident, shows her to have been of no ordinary strength of constitution.—(*Lancet*, August, 1860.)

IV.—*Retroflexion of the Unimpregnated Uterus.* By John Moir, M.D., F.R.C.P., Edinburgh.

Retroflexion occurs in two very opposite states of the uterus: first, in the normal unimpregnated state; and second, in the uterus soon after delivery, when its bulk and attachments are in a very different condition. For a long time he employed, in the treatment of these cases, the simple intra-uterine bougie as recommended by Professor Simpson, or the wire pessary of the same author, but, as annoying cases often occurred, where, after the removal of the instrument, the uterus resumed its abnormal position, he was compelled to resort to some other expedient. It occurred to him that if, by the introduction of a graduated series of sponge tents into the uterus, that organ would gradually dilate, as in a case of early miscarriage, and then allowed to contract over some well adapted instrument, the tendency to flexion might be overcome.

This plan was employed in several instances, and with the most complete success. The sponges must be of the smallest possible diameter. As considerable difficulty sometimes attends the introduction of the first sponge, from its softening in the vagina before it can be passed into the os and cervix, the operator must be satisfied then with getting it into the cervix the first day. The next day, little difficulty is experienced in passing a fresh one up to the fundus, but this must be done before proceeding with a larger one. After having passed it thus far, it should be allowed to remain one or two days, and a larger one then introduced, and so on till one, the thickness of the thumb, is introduced. From two to three weeks is required for this purpose. Care must be taken to suspend the treatment if much irritation is produced, and the symptoms properly treated ere a further trial is made. In only one case was he compelled to cease for a length of time, as severe hemorrhage was induced, to which, however, the patient was liable from slight causes. On removing the last sponge, an intra-uterine wire pessary, the stem of which is covered with gutta-percha to the size of about half an inch in diameter, is introduced into the cavity of the uterus, and allowed to remain for one or two days. This is replaced by another, with the gutta-percha about half as thick, and so on till the ordinary wire pessary is arrived at, and which remains for five or six days. After its removal, for a week or two the patient should remain in a recumbent posture. The uterus should be daily examined, and any flexion at once remedied. If all goes well, the patient should gradually return to her usual habits, but great care must be observed to keep the bowels easy, and avoid any straining or violent exertion.

As the sponge is so disagreeable to use, other remedies have been employed. India-rubber dilaters were tried, and seem likely to answer well.—(*Edinburgh Med. Journal*, February, 1860.)

V.—*Phlegmasia Dolens.* By W. TILBURY FOX, M.D., London.

This paper, read before the Obstetrical Society of London, regarded the view that the disease was dependent upon inflammatory changes in the vessels as too limited. The various states in which it occurs are: as the sole local disease, as after abortion, removal of polypi, etc.; as part of a general disease, either of virus origin, as puerperal fever, erysipelas, dysentery, etc., or abnormal nutrition, as cancer, phthisis, etc.; as complicating other diseases, as iliac abscess, suppressed menstruation, etc.

Its evolution depends upon the existence of obstructive disease in both veins and lymphatics. The causes of such obstruction might be classed as extrinsic or intrinsic in regard to the vessels, the former being comprised under the head of pressure from without; the latter, all producing coagulation, being subdivided under true phlebitis conjoined with angeio-leucitis, thrombosis, a mixture of these two, and preternatural coagulability of the blood.

It was insisted upon that this affection, except in its epidemic form, is in the majority of cases a pure thrombus, produced by sudden absorption of morbid fluid, the effect of sudden loss,—the class of cases in which it occurs, viz., diseases accompanied with breach of surface, as after labor, cancer, phthisis, the removal of polypi, being especially favorable to such an occurrence. This mode of production applies equally well to many cases of œdema, which pressure leaves unexplained, the difference between the two cases being, that in phlegmasia dolens the lymphatics are involved in addition to the veins.

Dr. Rigby remarked upon a case he had lately seen, occurring in the arm of a patient with a very feeble circulation. It was precisely one of those cases of coagulation in the veins described by Dr. Humphrey. He had followed the practice of Dr. Humphrey, of giving ammonia for the purpose of dissolving or breaking down these coagula, and with the best effects. There could be no doubt that the affection was frequently caused by the absorption of putrid matter.

Dr. Graily Hewitt observed that there were two questions for solution; the one being the *nature*, the other the *cause* of the pathological changes which occur in this disease. He conceived that they consisted in coagulation of the blood within the veins, and could not admit that the lymphatics were primarily or necessarily affected. The cause, he believed, might be produced by the action of septic matter introduced into the veins, as explained by the author. He would, however, explain the occurrence of coagulation in the veins, in the majority of cases coming under the notice of obstetricians, in another way. The influence of deficient contraction of the uterus after delivery was very important. The uterine sinuses remain open, become filled with coagula which spread upward, the iliac veins are invaded, and phlegmasia dolens occurs. When hemorrhage takes place, the same thing occurs. He believed that the disease would be rarely observed if steps were always taken to insure due contraction of the uterus after delivery.

Dr. Druitt regarded the affection as a member of that class of pathological exudations which includes also the diffuse inflammation of the areolar tissue, the phlegmonous erysipelas, the carbuncle, etc. A poison introduced into the areolor tissue, either locally or through the circulation at large, was sufficient to explain the phenomena. He considered it a retrogression to assert that obstruction was the cause. This will produce its mechanical effects, as œdema, but could not account for phlegmonous erysipelas or carbuncle. The true pathology of the disease was, that the imbibition of poison from a wound or ulcer produced not merely coagulation of blood in veins, but that yet undefined change in the relation of the blood and tissue which caused a peculiar exudation.

Dr. Barnes referred to the essays of Mr. Henry Lee and Dr. MacKenzie as admirable and complete contributions to the pathology of this malady. He did not consider that hemorrhage disposed to this affection by favoring imperfect contraction of the uterus, but by increasing the absorbing function. He, too, had found the treatment by ammonia the most useful.

Dr. Fox, in reply, could not disallow the lymphatics a share, and asked Dr. Hewitt to explain the difference in the character of the swelling in obliteration of the inferior vena cava and innominate vein, except upon the fact that the lymphatics in the latter case were obliterated. Again, Dr. Rigby's well-known case, where no disease but of the lymphatics existed, was surely sufficient to establish the participation of the lymphatics. Dr. Hewitt's explanation of the production of phlegmasia dolens would explain only the puerperal cases, not those of cancer, phthisis, etc.

In answer to Dr. Druitt, who denied the mechanical cause of the disease, he desired to know how this would apply to a case where a cancerous tumor presses on the iliacs, and phlegmasia dolens appeared with great pain, but when the tumor descended into the pelvis, and got up behind the bladder to the other side, the pressure being removed, the disease and pain disappeared at once.

All discussion turned upon the use of fibrin in the economy. Virchow, Zimmerman, Cappie, and Simon believe it to be a result of cellular tissue, and brought to the blood by the lymphatics. That fibrin is not excrementitious seemed evident from its use in the economy and capability of organization; its mode of production, namely, result of elaborative (digestive and glandular) act, and oxidation of albumen; its chemical composition, unlike waste products; its relation to the pale corpuscles, being increased or decreased in direct ratio with them; its relation to connective tissue as shown by Liebig, hence it is the pabulum of areolar tissues; in the lower animals we see corpuscles existing before fibrin in the blood,—in the articule series we get fibrin, and with it the colorless nucleated cell,—and seeing always that the pale corpuscles keep in the blood stream inside, it seems not unlikely, indeed certain, that the pale corpuscles are related to the tissues outside in preparing the fibrin for the connective tissues. Where areolar tissue is, there are lymphatics, and vice versa; hence there must be some close connection. It seems that when the arterial blood reaches the areolar tissues the latter draw upon it for pabulum, and the excess is removed by the lymphatics. Obstruct the lymphatic circulation, as in phlegmasia dolens, and hypertrophy of the cellular tissue and gelatinization in the areolæ ensue.—(*London Lancet*, August, 1860.)

### VI.—*The Influence of Slow Poisoning, by the Preparations of Lead, on the Product of Conception.* By CONSTANTINE PAUL, Paris.

From the valuable paper on this subject, we present the following summary of the researches of M. Paul, who, as an *interne* of the hospitals of Paris, has enjoyed ample opportunity for observation. Those women who handle type as polishers in founderies are most affected by saturnine diseases. He found, in a series of observations, four women had had fifteen ascertained pregnancies—of these ten ended in abortion, two in premature labor, one in still-birth, and one child lived but twenty-four hours.

In another series of cases, five women had an aggregate of nine children at term prior to exposure to the poisoning, and no accidents of pregnancy. Afterwards, they had thirty-six pregnancies, of which twenty-six aborted, one had premature labor, two ended in still-births, five children died, four in the first year, and two were living, one being puny and ailing, the other only three years old.

Another woman had five abortions, quitted the business, and bore a healthy child.

Another left the trade for two periods, bore in these intervals two healthy children, and, returning, had two abortions. The same influence was observed when the fathers were exposed. In seven cases, every woman had an abortion; of thirty-two pregnancies, twelve children were born prematurely. Of twenty living children, eight died in the first year, four in the second, five in the third, one after the third, and two remained.

In other observations, M. Paul showed that where the lead affection was less marked, there was a corresponding diminution of the effect upon the pregnancies.—(*Arch. Gén. de Méd.*, May, 1860.)

### VII.—*Fatal Effects Arising from the Enlargement of the Thymus Gland in Children.* By G. S. BRENT, L.S.A., London.

On several occasions a fatal result has been observed from the enlargement, or perhaps non-absorption, of the thymus gland in young children. One case was that of a child aged two and a half years, well nourished, and in every way a fine boy, who died suddenly, after a slight illness of two days. He had two spasms just before death, but no apparent difficulty of breathing. The post-mortem revealed no external indication of the cause, and internally every organ was found healthy and natural except the thymus gland, which was preternaturally large and vascular; it weighed an ounce and a quarter. The effect of this condition would probably be pressure upon the adjacent nerves to such an extent as to irritate them, and occasion a spasm of the glottis, to which event the death was attributed.

In a similar case, Mr. Hemming, of Notting Hill, found the enlarged gland pressing upon the right recurrent nerve of the par vagum. In this case, the child was erroneously supposed to have swallowed some hot water.

Dr. Hayne, of Camdentown, encountered a case where the child was twenty months old, and death occurred very suddenly. The thymus weighed ten drachms. Many other instances have occurred where death took place, apparently from spasmodic closure of the glottis, where considerable enlargement existed. In the human subject, this gland increases in size very rapidly after the seventh month of fœtal life, and at birth varies from eighty-four to two hundred and forty grains in weight. At nine months afterwards, it is found to be about two hundred and seventy grains, after which it generally declines in size.

Kopp has noticed several cases of suddenly fatal dyspnœa in children, where the gland was of large size, and concludes that there is some essential connection between this enlargement and the suffocation.

According to Copland, Mr. Hood, of Kilmarnock, was the first to direct attention to this matter. He does not, however, even hint that the effects are other than mechanical, such as pressure on the veins at the top of the chest, inducing congestion and effusion in the head; and does not appear to suppose that this unusual size is accompanied by an increased nervous susceptibility. A correlation evidently exists between the size of the thymus and the development of the brain and impressibility of the nervous system. It has been observed in these instances of sudden death coincident with abnormal enlargement of this gland, that the children have been peculiarly forward and intelligent for their age. In the Cyclopædia of Anatomy and Physiology it is stated that "absence of the gland has only been observed in cases of acephalism, where the brain and many other parts have been simultaneously deficient." If it be true, then, that enlargement of the thymus has any relation to the greater development of nervous force, there will be a double reason why an unusual size or a non-absorption of this body should produce suffocative dyspnœa, viz., both from the greater excitability produced, and the inordinate pressure exercised by the gland in a state of turgidity.—(*Lancet*, Sept. 8, 1860.)

### VIII.—*Treatment of Hooping-cough by Sulphate of Zinc and Extract of Belladonna.* By H. W. Fuller, M.D., London.

In this paper, read before the Harveian Society, the author alluded to the popular opinion that hooping-cough must run its course. This he combated by reference to the results of his own experience. His attention was directed to the matter by the inefficiency of the ordinary treatment, and the neglect of all measures to subdue the tendency to spasm. He regarded this affection as essentially one of bronchitic irritation, accompanied by reflex spasm of the air-passages, and believed the latter demanded the most serious attention, as it is not only painful, but may give rise to life-long mischief. He had found the administration of the sulphate of zinc, in rapidly increasing doses, to be the most successful method of subduing the spasm. He then referred to his discovery of the tolerance of belladonna by children, the condition being, that it should be given in divided doses at least four times daily, at first in small doses, which may be increased day by day, or on alternate days, by a corresponding amount. Dilatation of the pupil need not be regarded as a bar to its continuance, and if the precautions just noticed are observed, the daily dose of the extract of belladonna may be safely increased to a scruple or half a drachm without any unpleasant symptoms. These facts he brought to bear on the disease in question, and, from the conjoint use of sulphate of zinc and extract of belladonna, in rapidly increasing doses, he had obtained results highly beneficial. Rarely had he found the hoop to last above twenty-one days, and in some instances it had subsided in ten days. He gives the combination as soon as the hoop declares itself. If the attack is accompanied by much febrile excitement and bronchitic irritation, he prescribes a mixture of a drachm each of antimonial wine and wine of ipecacuanha to two ounces of water, of which a larger or smaller amount is given, according to circumstances. If necessary, he applies a blister to the chest. In all cases, however, the belladonna and zinc are perseveringly given. To children under three years of age, he begins by employing one-sixth of a grain of extract of belladonna and half a grain of sulphate of zinc four times a day, and to those above that age, a quarter of a grain of the former and a grain of the latter. The remedies are given in solution in water, and the dose of each substance is increased by a corresponding dose daily, or on alternate days, so that the child who began by taking a quarter of a grain of the extract and one grain of zinc at a dose, would be taking one grain of the extract and four grains of zinc at a dose either on the fourth, sixth, or eighth day, according to the rapidity with which the dose is increased.—(*Lancet*, July 28, 1860.)

IX.—*Convulsions in Children Treated with the Ethereal Tincture of Valerian.* By W. HARDEE, M.D., Savannah.

No claim is made by Dr. H. for the position of a discoverer, but having found this remedy highly beneficial in several cases, he is anxious to attract the attention of the profession to it. The cases were all from one to three years of age. He employs it in doses of four to six drops every fifteen to thirty minutes, until all twitching ceases, after which it may be continued according to circumstances along with other remedies for the particular indications. It may also be employed at the same time in the form of enema, say forty to sixty drops in flaxseed tea or water, the dose being graduated to the age and symptoms of the patient.—(*Atlanta Med. and Surg. Jour.*, Sept., 1860.)

---

